# The epidemiology and burden of atherosclerotic cardiovascular disease in China from 1990 to 2021: findings from the global burden of disease 2021

**DOI:** 10.3389/fpubh.2025.1529506

**Published:** 2025-06-26

**Authors:** Xin-Zheng Hou, Qian Wu, Ying-Tian Yang, Xue-Jiao Ye, Qian-Yu Lv, Chen-Yan Yang, Ming-Yu Huang, Shi-Han Wang

**Affiliations:** Department of Cardiovascular Diseases, Guang'anmen Hospital, China Academy of Chinese Medical Sciences, Beijing, China

**Keywords:** ischemic heart disease, stroke, peripheral arterial disease, China, disease burden

## Abstract

**Background:**

Atherosclerotic cardiovascular disease (ASCVD) significantly threatens the health of the Chinese population. Understanding its epidemiological burden is vital for targeted interventions.

**Methods:**

Using Global Burden of Diseases (GBD) 2021 data, we assessed Disability-Adjusted Life Years (DALYs), incidence, prevalence, and mortality of ischemic heart disease (IHD), stroke, and lower extremity peripheral artery disease (PAD) in China in 2021. Joinpoint regression analyzed trends from 1990 to 2021, and risk factor contributions to the disease burden were evaluated.

**Findings:**

In 2021, age-standardized rates (ASRs) per 100,000 population for IHD (DALYs, incidence, prevalence, mortality) were 1856.5 (1548.7, 2159.8), 365.7 (293.3, 440.1), 3042.3 (2601.7, 3629.9), and 110.9 (92.4, 128.6), respectively, with males bearing a higher burden. For stroke, ASRs were 2648 (2253.4, 3076.9), 204.8 (181, 231.5), 1301.4 (1200.6, 1405.7), and 138 (116.7, 160.3), also higher in males. Lower extremity PAD showed ASRs of 8.4 (4.9, 14.3), 112.7 (97.8, 130.7), 1331.1 (1147.5, 1544.2), and 0.1 (0.1, 0.2), with a higher burden in females. Metabolic risks had the largest population-attributable fraction.

**Conclusion:**

As of 2021, the epidemiological burden of IHD, stroke, and PAD in China remains substantial, with notable gender disparities. Metabolic risks significantly contribute to this burden.

## Introduction

Atherosclerotic cardiovascular diseases (ASCVDs) are the leading cause of mortality worldwide, imposing substantial epidemiological burdens. In 2019, ischemic heart disease (IHD) was responsible for approximately 9.14 million of all global deaths, with a disproportionate burden in low- and middle-income countries (1). In China, IHD and stroke resulted in 1,736,000 and 2,098,000 deaths, respectively, in 2016 (2). The prevalence of carotid artery atherosclerotic plaque among Chinese adults reached 21% (3). Additionally, there is a notable socioeconomic inequality in the burden of ASCVD in China (4). All of the above reflects an alarming public health crisis that challenges healthcare systems and economic stability. ASCVDs encompass various conditions such as IHD, stroke, and peripheral artery disease (PAD), all of which share a common pathological mechanism of atherosclerosis, thereby presenting overlapping opportunities for prevention strategies (5). While recent initiatives in China have led to significant reductions in the burden of certain cardiovascular events, the continued epidemiological burden of these diseases remains a critical concern; reports indicate that the incidence of IHD in China is still on the rise from 1990 to 2019 (6). Thus, understanding the latest epidemiological trends of ASCVD is crucial for tailoring effective preventive strategies that can address this ongoing public health challenge and improve health outcomes.

ASCVD arises from the complex interplay between genetic susceptibility and a variety of risk factors (7). Understanding the contribution of risks to the disease burden is crucial for developing effective prevention strategies. For instance, a study involving 791,373 Chinese adults found that approximately 49,861,000 cardiovascular deaths between 2010 and 2018 could be attributed to elevated fasting blood glucose levels (8). Consequently, strategies to reduce the disease burden through the management of fasting blood glucose were proposed. By quantifying the attributable fractions of risks, public health interventions can be prioritized, targeting the most significant causes to mitigate the rising burden. With the intensification of aging and demographic shifts, it is of practical significance to promptly update the disease burden of ASCVD for the formulation of public health policies. In June 2024, the Institute for Health Metrics and Evaluation (IHME) at the University of Washington released the latest global burden of disease data, which serves as a crucial tool for understanding global disease characteristics. However, there have been scant reports on the detailed micro-epidemiological burden of ASCVD and risks in China based on this updated dataset. Therefore, we conducted this study.

Using the Global Burden of Diseases (GBD) data, we reported the numbers and rates of disability-adjusted life years (DALYs), incidence, prevalence, and deaths for IHD, stroke, and PAD in China in 2021. We analyzed the contributions of environmental, behavioral, and metabolic risks to the disease burden. Joinpoint analysis was employed to explore trends in the disease burden from 1990 to 2021, and subgroup analyses were conducted based on gender and age groups. Our findings provide critical insights into the current ASCVD disease burden in China.

## Methods

### Data source

The data was sourced from the GBD. The GBD is the largest and most comprehensive global research endeavor to quantify health levels and the burden of diseases, led by the IHME at the University of Washington (9). In June 2024, GBD released the latest data, which includes information up to 2021, covering 204 countries and regions, 371 diseases and injuries, and 88 risk factors (10). The GBD estimation in China was derived from multiple data sources, including disease surveillance points of the China CDC, death registration systems, and vital registration systems in Hong Kong and Macao (11). This study adhered to the guidelines for Accurate and Transparent Health Estimates Reporting: the GATHER statement (12). The data can be accessed at the following website: https://vizhub.healthdata.org/gbd-results/. The downloaded GBD 2021 estimate was utilized in our analysis, and no re-estimation was conducted.

### Diseases definition

In this study, ASCVD referred to IHD, stroke, and lower extremity PAD. The diagnostic criteria were based on the International Classification of Diseases (ICD-9 and ICD-10). The corresponding ICD-10 codes were I20-I25.9, and the ICD-9 codes were 410–414.9 for IHD. Stroke referred to ischemic stroke, cerebral hemorrhage, and subarachnoid hemorrhage. For strokes, the ICD-10 codes were G45-G46.8, I60-I63.9, I65-I66.9, I67.0-I67.3, I67.5-I67.6, I68.1-I68.2, and I69.0-I69.3, while the ICD-9 codes were 430–435.9, 437.0–437.2, and 437.5–437.8. The ICD-10 codes for lower extremity PAD were I70.2-I70.8 and I73-I73.9, and the ICD-9 codes were 440.2, 440.4, and 443.0–443.9.

### Epidemiological burden of disease

DALYs, incidence, prevalence, and deaths were examined. The DALYs represent the sum of Years of Life Lost (YLL) and Years Lived with Disability (YLD) (13). The YLL refers to the life expectancy foregone due to premature death and is calculated by subtracting the age at death from the maximum life expectancy for that age group. The YLD represents the years lived in less than full health due to disease, measured by multiplying the prevalence of the disease by its disability weight. The disability weight indicates the severity of the disease, ranging from 0 (perfect health) to 1 (equivalent to death). The incidence refers to the occurrence of new cases of a particular disease within a specified population over a certain period. The prevalence represents all cases (both new and old) of a disease within a population at a specific point in time. DisMod-MR2.1 and standard CODEm methods were used to estimate DALYs, incidence, prevalence, and deaths.

### Risk factor attribution analysis

To evaluate the attribution of risks to the disease burden, population-attributable fractions (PAF) were employed. Exposure data were modeled using spatiotemporal Gaussian process regression or DisMod-MR 2.1 to generate quantitative relative risk estimates for each risk-outcome pair. These estimates were then paired with corresponding exposure estimates to calculate the PAF for each risk-outcome pair. The PAF was multiplied by the transition rates to determine the attributable DALYs. Previous studies have described the above calculation process (14). We investigated 12 risks associated with ASCVD, which can be categorized into 3 groups: environmental, behavioral, and metabolic risk factors. Environmental risks included air pollution, non-optimal temperature, and other environmental risks. Behavioral risks encompass tobacco, low physical activity, high alcohol consumption, and dietary risks. Metabolic risks consisted of high low-density lipoprotein (LDL) cholesterol, high systolic blood pressure, high fasting plasma glucose, high body mass index, and kidney dysfunction.

### Statistical analysis

We reported the numbers, crude rates (CR), and age-standardized rates (ASR) per 100,000 population related to DALYs, incidence, prevalence, and deaths in 1990 and 2021. The ASR was calculated based on the global population age structure in 2021. The total percentage change between the 2 years was calculated. Furthermore, the 95% uncertainty intervals (UI) for estimates were reported. In the GBD, estimate was calculated 1,000 times, with sampling from distributions in each iteration. The 95% UI was determined by the 25th and 975th values in the ordered sequence of these 1,000 values.

For subsequent analyses, we focused on DALYs and incidence. We visualized the numbers and CRs for different genders and age in 2021. To examine the trends, we visualized the numbers and ASRs for males and females from 1990 to 2021. To quantify trends, Joinpoint regression analyses were conducted. We used the BIC value to select the optimal number of nodes and applied log-linear models to calculate the annual percentage change (APC). The average annual percentage change (AAPC) was further calculated based on the APCs of each segment and their weights. If the AAPC and its 95% confidence interval were both greater than 0, it indicated an overall upward trend; otherwise, it indicated a downward trend. We reported the percentage attributable contribution of risks to age-standardized DALYs. Above analyses were performed using R (Version 4.4.1) and Joinpoint (Version 5.2.0) statistical software.

## Results

### Disease burden in 1990 and 2021

The numbers, CR, and ASR of DALYs, incidence, prevalence, and deaths for IHD, stroke, and lower extremity PAD were presented in Table 1.

**Table 1:** Disease burden of ASCVDs in 1990 and 2021.

Matrix	1990			2021			1990–2021
	Number (n, UI)	CR (per 100,000, UI)	ASR (per 100,000, UI)	Number (n, UI)	CR (per 100,000, UI)	ASR (per 100,000, UI)	Total percent change of ASR (%, UI)
All genders							
Ischemic heart disease							
DALYs	13624111.9 (12056605.6, 15466092.3)	1158.1 (1024.8, 1314.6)	1771.1 (1574.8, 1990.7)	35672627 (29920272.8, 41738945.8)	2507.3 (2103, 2933.7)	1856.5 (1548.7, 2159.8)	5 (−14, 28)
Incidence	2301643.5 (1861968.5, 2792193.4)	195.6 (158.3, 237.3)	315.3 (255.5, 382.5)	7304573.2 (5815313.2, 8949994.7)	513.4 (408.7, 629.1)	365.7 (293.3, 440.1)	16 (14, 18)
Prevalence	19505463.1 (16754811.3, 22537174)	1658 (1424.2, 1915.7)	2526.4 (2190, 2915)	63331311.5 (53, 812, 323.8, 76, 196, 537.0)	4451.3 (3782.3, 5355.6)	3042.3 (2601.7, 3629.9)	20 (14, 28)
Deaths	547845.1 (486106.5, 617005.7)	46.6 (41.3, 52.5)	94.1 (84, 105.9)	1956859.4 (1634477.6, 2280131.2)	137.5 (114.9, 160.3)	110.9 (92.4, 128.6)	18 (−2, 41)
Stroke							
DALYs	38003356.6 (33428261.5, 42843504.8)	3230.3 (2841.4, 3641.7)	4834.8 (4242.6, 5418.8)	53190691.2 (45108715.8, 61958023.9)	3738.6 (3170.5, 4354.8)	2648 (2253.4, 3076.9)	-45 (−54, -34)
Incidence	1685761.8 (1506913.7, 1897237.4)	143.3 (128.1, 161.3)	226.9 (202.9, 252.8)	4090480.1 (3593818.7, 4699827.9)	287.5 (252.6, 330.3)	204.8 (181, 231.5)	−10 (−15, -4)
Prevalence	10731080.1 (10003053.9, 11542610.1)	912.1 (850.3, 981.1)	1167.4 (1082, 1262.6)	26335402.6 (24154963, 28625610.9)	1851 (1697.8, 2012)	1301.4 (1200.6, 1405.7)	11 (8, 14)
Deaths	1530590.4 (1334889.3, 1721546.8)	130.1 (113.5, 146.3)	242.2 (213.8, 272.7)	2591646.9 (2179395.8, 3032694.9)	182.2 (153.2, 213.2)	138 (116.7, 160.3)	−43 (−53, -31)
Lower extremity peripheral arterial disease							
DALYs	63347.8 (35290.5, 112726.5)	5.4 (3, 9.6)	9 (5.1, 16)	171757.2 (99158.2, 301527.7)	12.1 (7, 21.2)	8.4 (4.9, 14.3)	−7 (−16, 6)
Incidence	923622.3 (796818.5, 1085328.7)	78.5 (67.7, 92.2)	109.6 (94.8, 127.1)	2447371.7 (2109991.3, 2854291)	172 (148.3, 200.6)	112.7 (97.8, 130.7)	3 (2, 4)
Prevalence	9818058.8 (8300902.5, 11519182.3)	834.5 (705.6, 979.1)	1250.8 (1082.2, 1447)	28474885.9 (24446552.7, 33220180.3)	2001.4 (1718.3, 2334.9)	1331.1 (1147.5, 1544.2)	6 (5, 8)
Deaths	569 (419.5, 717.6)	0 (0, 0.1)	0.1 (0.1, 0.1)	2332.4 (1807.7, 2953.6)	0.2 (0.1, 0.2)	0.1 (0.1, 0.2)	26 (−13, 87)
Males							
Ischemic heart disease							
DALYs	7556607.8 (6300651.2, 8936137.9)	1245.2 (1038.3, 1472.6)	2044.5 (1742.5, 2379.7)	21529833.9 (16894341.6, 26751533)	2957 (2320.3, 3674.1)	2478 (1979.9, 3018)	21 (−8, 57)
Incidence	1244366.2 (991819, 1520020.3)	205.1 (163.4, 250.5)	348.6 (280, 423.6)	3818580.2 (3034880.7, 4650551.5)	524.5 (416.8, 638.7)	401.2 (322, 481.2)	15 (12, 18)
Prevalence	10648703 (9142879.9, 12348341.1)	1754.8 (1506.6, 2034.9)	2838.2 (2466.6, 3277.4)	33571871.7 (28288613.4, 40380503.2)	4610.9 (3885.2, 5546)	3379.2 (2895.9, 4036.3)	19 (13, 26)
Deaths	280998.7 (235025, 331060.1)	46.3 (38.7, 54.5)	109.8 (95, 125.4)	1088196.6 (868768.3, 1332607.6)	149.5 (119.3, 183)	148.4 (121.2, 179)	35 (6, 72)
Stroke							
DALYs	20613874.7 (17164482.2, 24105597.9)	3396.9 (2828.5, 3972.3)	5528.9 (4702.3, 6366.4)	31862593.3 (25731004.2, 39019269)	4376.1 (3534, 5359)	3444.8 (2818.1, 4184.1)	−38 (−52, -20)
Incidence	900160.9 (802269.9, 1027867.6)	148.3 (132.2, 169.4)	251.6 (224.7, 283)	2305941.6 (2016192, 2658470.7)	316.7 (276.9, 365.1)	240.9 (212, 274.9)	−4 (−11, 2)
Prevalence	5321447.3 (4946271.7, 5762679.6)	876.9 (815.1, 949.6)	1205.9 (1112.5, 1315)	13719379 (12615636.6, 14899172.7)	1884.3 (1732.7, 2046.3)	1385.9 (1282.7, 1498.3)	15 (11, 19)
Deaths	796977.4 (669509.1, 926817.3)	131.3 (110.3, 152.7)	284 (243.8, 323.3)	1506012.2 (1216251.7, 1855990.7)	206.8 (167, 254.9)	186.8 (152.4, 226.9)	−34 (−49, -16)
Lower extremity peripheral arterial disease							
DALYs	19374 (12087.2, 31229)	3.2 (2, 5.2)	5.8 (3.6, 9.4)	57008.7 (37840.1, 86958.9)	7.8 (5.2, 11.9)	6.1 (4.1, 9.1)	4 (−17, 40)
Incidence	262877.5 (223005.1, 312197.6)	43.3 (36.8, 51.5)	63.5 (55.2, 73.8)	706399.8 (607065.5, 830957.2)	97 (83.4, 114.1)	67.5 (58.7, 78.5)	6 (5, 8)
Prevalence	2636098.9 (2221733.4, 3099360.9)	434.4 (366.1, 510.7)	679.5 (582.8, 785.6)	7684182 (6524262.6, 9027019.4)	1055.4 (896.1, 1239.8)	747.8 (641.6, 868.5)	10 (8, 12)
Deaths	314.1 (217.5, 429.8)	0 (0, 0.1)	0.1 (0.1, 0.2)	1371.1 (962.6, 1850.9)	0.2 (0.1, 0.2)	0.2 (0.1, 0.2)	39 (−18, 139)
Females							
Ischemic heart disease							
DALYs	6067504 (5114360.2, 7253344.5)	1065.2 (897.9, 1273.4)	1548.1 (1305.2, 1839.2)	14142793.1 (11236414.5, 17447300.4)	2036 (1617.6, 2511.7)	1350.6 (1068.6, 1666.8)	−13 (−33, 11)
Incidence	1057277.3 (852791.6, 1292713.6)	185.6 (149.7, 226.9)	282.2 (229.4, 344.5)	3485993 (2792002.6, 4259778.4)	501.8 (401.9, 613.2)	328.1 (264, 397.2)	16 (13, 19)
Prevalence	8856760.1 (7651262.7, 10222044.4)	1554.9 (1343.2, 1794.5)	2235.3 (1938.3, 2585)	29759439.8 (25359422.4, 35857848.6)	4284.1 (3650.7, 5162.1)	2724.2 (2320.2, 3259.1)	22 (16, 30)
Deaths	266846.4 (223802.6, 318704.8)	46.9 (39.3, 56)	84.4 (71.2, 99.8)	868662.8 (676758.8, 1073177.6)	125 (97.4, 154.5)	86.1 (67.1, 106.4)	2 (−21, 28)
Stroke							
DALYs	17389481.9 (14747813.9, 20537192.4)	3052.8 (2589.1, 3605.4)	4283 (3647.2, 5018.3)	21328097.8 (17393679, 25769283.8)	3070.4 (2504, 3709.7)	1990.9 (1621.6, 2399.7)	−54 (−63, -41)
Incidence	785600.9 (700501, 879300.6)	137.9 (123, 154.4)	204.6 (182.5, 227.1)	1784538.6 (1562523.4, 2028532.9)	256.9 (224.9, 292)	170.1 (150.1, 191.1)	−17 (−22, -11)
Prevalence	5409632.8 (5041940.4, 5826048.1)	949.7 (885.1, 1022.8)	1149.6 (1064.7, 1241.7)	12616023.7 (11451737, 13793510.6)	1816.2 (1648.6, 1985.7)	1218.3 (1115.1, 1324.6)	6 (2, 10)
Deaths	733613 (616868.4, 863847.8)	128.8 (108.3, 151.7)	214.1 (178.9, 251.3)	1, 085, 634.7 (854, 437.5, 1, 336, 345.6)	156.3 (123, 192.4)	103.7 (81.6, 127.6)	−52 (−62, -37)
Lower extremity peripheral arterial disease							
DALYs	43973.8 (23169.4, 81283.9)	7.7 (4.1, 14.3)	11.5 (6.2, 21.2)	114748.4 (60713.9, 215455.7)	16.5 (8.7, 31)	10.3 (5.5, 19.1)	−10 (−17, -2)
Incidence	660744.8 (570298, 769994.3)	116 (100.1, 135.2)	153.1 (133, 177.6)	1740972 (1500126.4, 2028606.9)	250.6 (216, 292)	155.6 (134.9, 180.3)	2 (0, 3)
Prevalence	7181959.9 (6096852.2, 8388399.1)	1260.8 (1070.3, 1472.6)	1747.9 (1505.4, 2028.7)	20790703.8 (17849889.9, 24184370.6)	2993 (2569.7, 3481.6)	1854.9 (1596.9, 2148.6)	6 (5, 8)
Deaths	254.9 (184.6, 338.9)	0 (0, 0.1)	0.1 (0.1, 0.1)	961.3 (717.4, 1216.6)	0.1 (0.1, 0.2)	0.1 (0.1, 0.1)	10 (−28, 63)

CR, crude rates; ASR, age-standardized rate; UI, uncertainty intervals; DALYs, disability-adjusted life years.

#### IHD

The ASR of DALYs showed a significant upward trend, rising from 1,771.1 (1,574.8, 1,990.7) in 1990 to 1,856.5 (1,548.7, 2,159.8) in 2021. Gender differences were observed. The incidence and prevalence exhibited upward trends, with no significant gender differences. In 2021, the ASR of incidence for the total population, males, and females were 365.7 (293.3, 440.1), 401.2 (322, 481.2), and 328.1 (264, 397.2) respectively. In 2021, the ASR of prevalence for the total population, males, and females were 3042.3 (2601.7, 3629.9), 3379.2 (2895.9, 4036.3), and 2724.2 (2320.2, 3259.1) respectively. The death rates and trends exhibited significant gender specificity. In 2021, the ASR of death for the total population, males, and females were 110.9 (92.4, 128.6), 148.4 (121.2, 179), and 86.1 (67.1, 106.4), respectively.

#### Stroke

The ASR of DALYs in 1990 and 2021 were 4,834.8 (4,242.6, 5,418.8) and 2,648 (2,253.4, 3,076.9), respectively, indicating a downward trend of−45% (−54, −34%). Notably, the decline was more pronounced among females compared to males. The ASR of incidence were 204.8 (181, 231.5) in 2021, showing a decreasing trend. The decline among females was much greater than that among males. Conversely, the prevalence of stroke showed an upward trend. In 2021, the ASR of prevalence in the total population, males, and females were 1,301.4 (1,200.6, 1,405.7), 1,385.9 (1,282.7, 1,498.3), and 1,218.3 (1,115.1, 1,324.6), respectively, with increases of 11% (8, 14%), 15% (11, 19%), and 6% (2, 10%). By 2021, the ASR of deaths had declined to 138 (116.7, 160.3).

#### Lower extremity PAD

The DALYs attributed to lower extremity PAD were 63,347.8 (35,290.5, 112,726.5) in 1990 and 171,757.2 (99,158.2, 301,527.7) in 2021, whereas the ASR of DALYs were only 9 (5.1, 16) and 8.4 (4.9, 14.3), respectively. Notably, in 2021, the ASR of DALYs among females was higher than that among males. The ASR of incidence, prevalence, and mortality exhibited upward trends, with total percentage changes being 3% (2, 4%), 6% (5, 8%), and 26% (−13, 87%), respectively.

### Disease burden in subgroups in 2021

#### IHD

**Figure 1:**
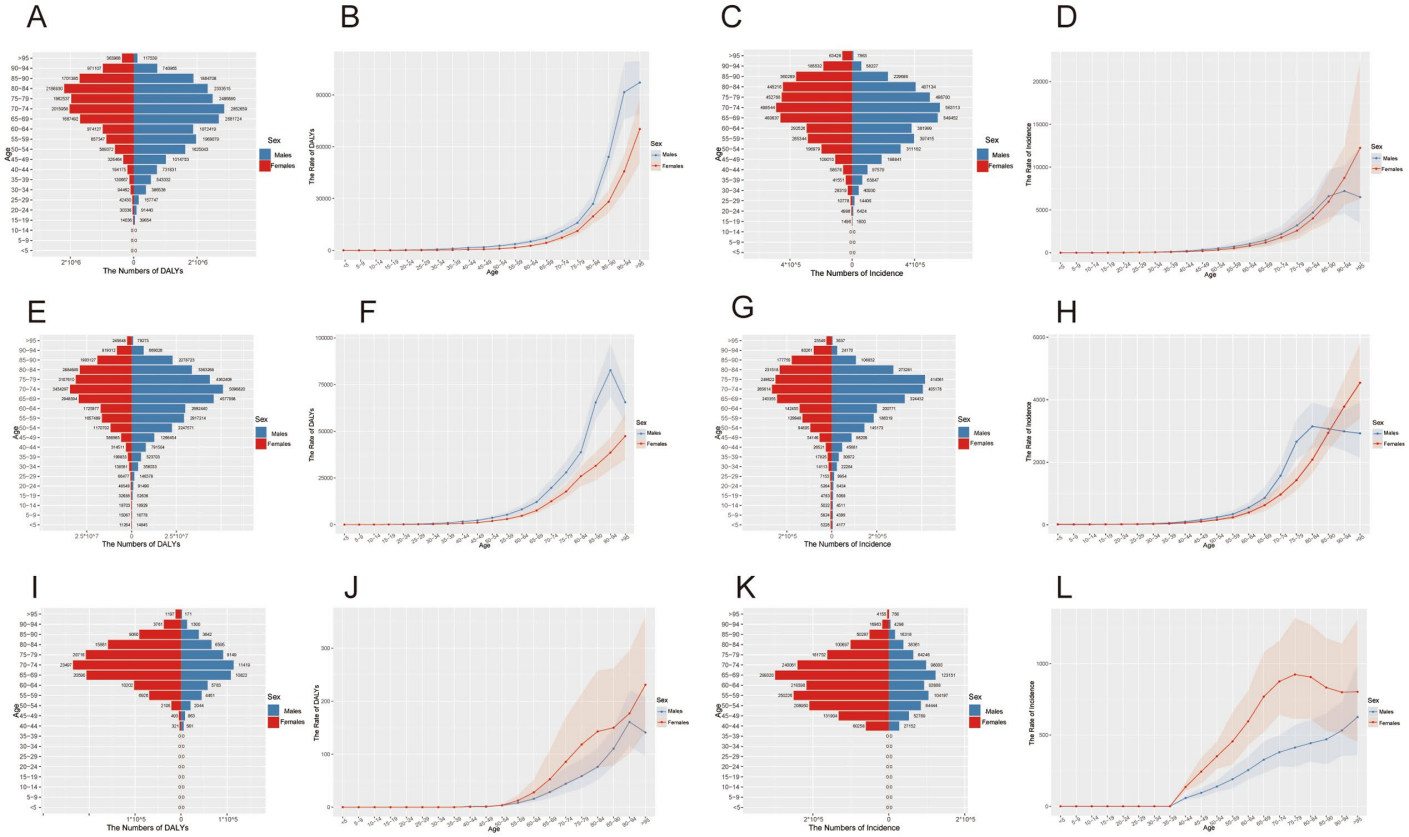
Gender and age characteristics of ASCVDs disease burden in 2021. **(A,E,I)** Number of DALYs for ischemic heart disease, stroke, and lower extremity peripheral arterial disease; **(B,F,J)** crude rate of DALYs per 100,000 population for ischemic heart disease, stroke, and lower extremity peripheral arterial disease; **(C,G,K)** number of incidence for ischemic heart disease, stroke, and lower extremity peripheral arterial disease; **(D,H,L)** crude incidence rate per 100,000 population for ischemic heart disease, stroke, and lower extremity peripheral arterial disease.

The peak in DALYs among female patients was observed in the 80–84 age group, approximating 2,186,930 (2,663,940, 1,722,810), whereas for males, the peak occurred in the 70–74 age group, at around 2,852,660 (3,601,811, 2,221,804). Across different age groups, the CR of DALYs was consistently higher in males than in females. Regarding incidence, the peak for both females and males was in the 70–74 age group, with values of 488,544 (686,737, 335,193) and 563,113 (787,801, 385,458), respectively. After the age of 90, the CR of incidence in females surpassed that in males. These findings were presented in Figures 1A–D.

#### Stroke

The peak in DALYs was observed in the 70–74 age group for both genders, with values of 5,096,820 (6,285,256, 4,043,519) for males and 3,434,297 (4,122,761, 2,785,104) for females. Across different age groups, the CR of DALYs was consistently higher in males than in females. The highest number of incident cases among females occurred in the 70–74 age group, amounting to 265,614 (346,914, 201,672), while for males was in the 75–79 age group, reaching 405,179 (506,345, 318,792). After the age of 85, the CR of incidence in females surpassed that in males. These results were presented in Figures 1E–H.

#### Lower extremity PAD

The peak in DALYs was observed in the 70–74 age group, with females recording 23,497 (46,339, 11,527), which was significantly higher than the 11,419 (19,421, 6,996) recorded for males. The peak incidence occurred in the 65–69 age group, where females had 299,320 (420,139, 202,868) incident cases, far exceeding the 123,151 (171,204, 87,256) in males. Across different age groups, the CR of both DALYs and incidence were consistently higher in females than in males. These findings were presented in Figures 1I–L.

### Trends in disease burden from 1990 to 2021

#### IHD

**Figure 2:**
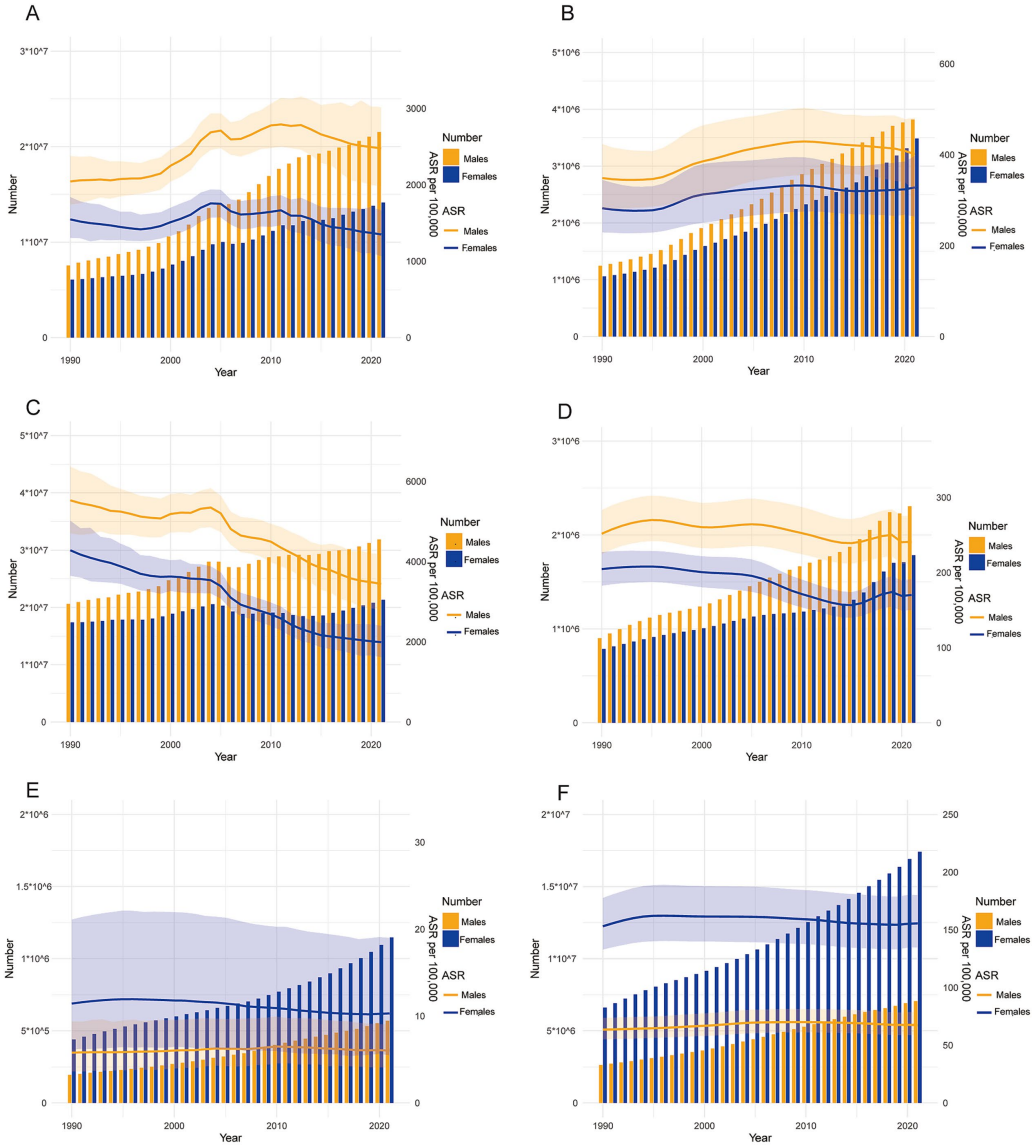
Trends in the number and ASR of incidence and DALYs for ASCVDs from 1990 to 2021. **(A,C,E)** Trends in the number and ASR of DALYs for ischemic heart disease, stroke, and lower extremity peripheral arterial diseases; **(B,D,F)** trends in the number and ASR of incidence for ischemic heart disease, stroke, and lower extremity peripheral arterial diseases.

**Figure 3:**
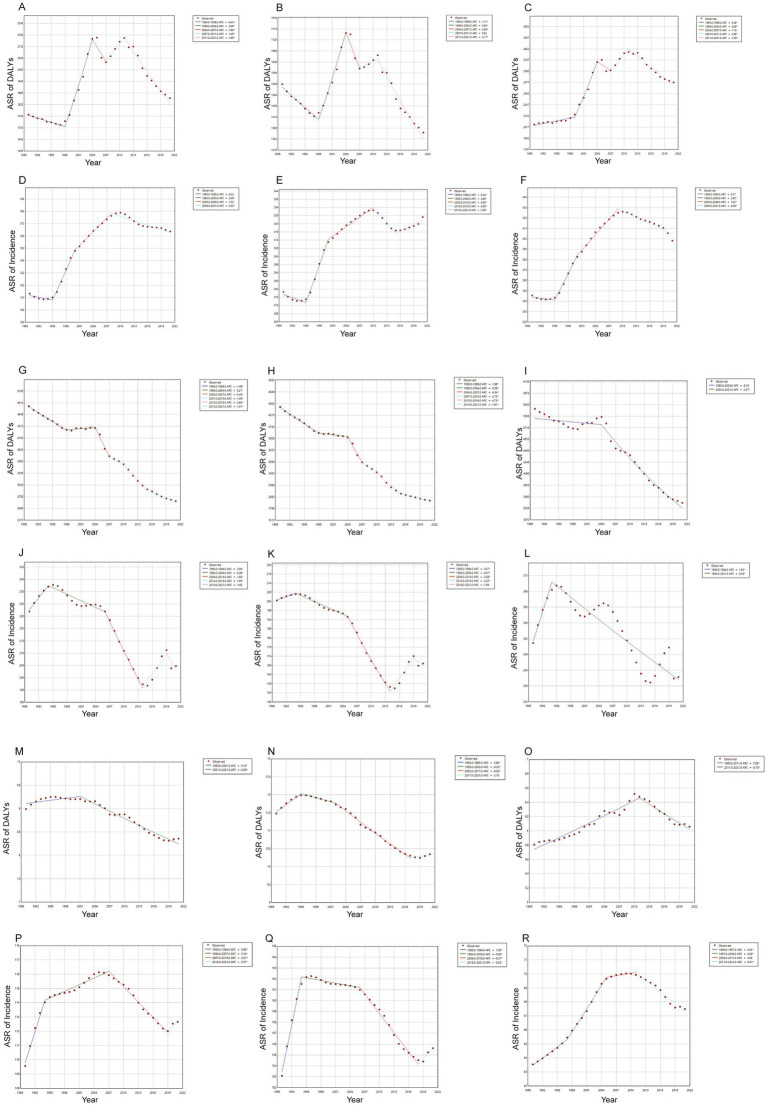
Trends in ASR of incidence and DALYs for ASCVDs from 1990 to 2021. **(A–C)** Trends in ASR of DALYs for ischemic heart disease in the general population, females, and males, **(D–F)** trends in ASR of incidence for ischemic heart disease in the general population, females, and males, **(G–I)** trends in ASR of DALYs for stroke in the general population, females, and males; **(J–L)** trends in ASR of incidence for stroke in the general population, females, and males; **(M–O)** trends in ASR of DALYs for lower extremity peripheral arterial diseases in the general population, females, and males, **(P–R)** trends in ASR of incidence for lower extremity peripheral arterial diseases in the general population, females, and males.

The DALYs consistently increased over the years in both genders, while the ASR of DALYs exhibits irregular fluctuations. These findings were illustrated in Figure 2A. Figures 3A–C presented segmented analyses of the ASR of DALYs across the entire population, females, and males, respectively. The AAPC of Figure 3A was 0.11 (−0.09, 0.32), indicating no overall linear trend in the entire population. The AAPC in Figure 3B was-0.48 (−0.71, −0.24), demonstrating that the ASR of DALYs among females exhibited an overall declining trend. Conversely, the AAPC in Figure 3C was 0.60 (0.37, 0.84), indicating an overall increasing trend among males. The incidence consistently increased over the years in both males and females, whereas the ASR of incidence seemed to exhibit an initial increase followed by a decline, as shown in Figure 2B. The AAPCs of Figures 3D–F were 0.49 (0.41, 0.57), 0.48 (0.42, 0.55), and 0.52 (0.42, 0.62) respectively, indicating an upward trend of incidence.

#### Stroke

The trend in DALYs due to stroke was presented in Figure 2C, with a continuous increase observed in males and a relatively stable pattern in females. However, the ASR of DALYs exhibited a declining trend across both genders. The AAPCs in Figures 3G–I were-1.93 (−1.80, −2.07), −2.46 (−2.59, −2.33), and-1.50 (−1.67, −1.33), respectively. The number of incidences continued to increase, while the ASR of incidence exhibited irregular variations, as shown in Figure 2C. The AAPC in Figures 3J,K were-0.36 (−0.52, −0.21) and −0.62 (−0.74, −0.50), respectively, suggesting the declining trend across the entire population and females. The AAPC in Figure 3L was-0.16 (−0.35, 0.03), indicating no linear trend among males.

#### Lower extremity PAD

Between 1990 and 2021, both the DALYs and incidence numbers related to lower extremity PAD consistently increased, as presented in Figures 2E,F. The AAPCs in Figures 3M,N were −0.32 (−0.38, −0.26) and −0.36 (−0.41, −0.30), respectively, indicating an overall declining trend of DALYs in the entire population and females. The AAPC in Figure 3O was 0.15 (0.09, 0.22), suggesting an overall increasing trend among males. Regarding the ASR of incidence, the increasing trend was observed in the entire population, females, and males. The AAPCs in Figures 3P,R were 0.09 (0.06, 0.11), 0.04 (0.02, 0.07), and 0.19 (0.16, 0.22), respectively.

### Risk factors analysis

The percentage contributions of risks to age-standardized DALYs were presented in Table 2. In 2021, metabolic risks contributed the largest epidemiological burden to IHD, stroke, and lower extremity PAD, with percentages of 74.9% (66.7, 81.7%), 67.7% (55.9, 76.9%), and 51.3% (41.9, 60.2%) respectively. For IHD and stroke, high systolic blood pressure accounted for the highest disease burden. For lower extremity PAD, high fasting plasma glucose was the primary contributor. Compared to 1990, the disease burden attributed to metabolic factors increased. For IHD, air pollution was the primary environmental risk, accounting for 34.5% (26.9, 42) in 2021. Although the impact of air pollution on stroke decreased significantly, at 29.9% (23.5, 37.7%) in 2021, it remained the primary environmental risk. The primary behavioral risk for stroke and lower extremity PAD was tobacco, accounting for 22.3% (18.3, 26.3%) and 26.8% (20.7, 33.1%) in 2021.

**Table 2:** Percentage contribution of risk factors to the disease burden of ASCVDs in 1990 and 2021.

Risks	Ischemic heart disease		Stroke		Lower extremity peripheral arterial disease	
	1990	2021	1990	2021	1990	2021
Environmental/occupational risks	51.8 (42.9, 59.7)	43.8 (35.4, 51.6)	55.1 (47, 62.8)	40.6 (32, 49.4)	1 (−0.1, 2.6)	0.9 (−0.1, 2.3)
Air pollution	43.9 (34.8, 52.2)	34.5 (26.9, 42)	46.9 (39.3, 54.5)	29.9 (23.5, 37.7)	/	/
Non-optimal temperature	7.8 (6, 10.1)	7.6 (6.1, 10.3)	8.1 (6.8, 10.2)	7.6 (6.3, 9.6)	/	/
Other environmental risks	6.8 (−0.9, 14.7)	7.2 (−1, 15.1)	8.4 (−1.1, 18.6)	8.4 (−1.1, 18.7)	1 (−0.1, 2.6)	0.9 (−0.1, 2.3)
Behavioral risks	67.8 (36, 84.7)	59.6 (31.4, 77.8)	43.5 (34.3, 53.5)	40.5 (30.1, 52)	37.2 (30.1, 44.9)	34.1 (27, 41.9)
Tobacco	32.5 (27.6, 37.5)	29.1 (24.2, 33.9)	24.4 (20.2, 28.3)	22.3 (18.3, 26.3)	30.7 (24.2, 37.6)	26.8 (20.7, 33.1)
Low physical activity	2.3 (1, 4)	2.5 (1, 4.3)	1.3 (0.3, 2.6)	1.9 (0.2, 4)	3.1 (0.9, 6.1)	3.2 (0.9, 6.5)
High alcohol use	−2.5 (−4.1, -0.5)	−2.1 (−3.4, -0.4)	5 (1.1, 9.4)	6.5 (1.6, 12.2)	/	/
Dietary risks	54 (4.3, 79.3)	44.2 (1.5, 70.1)	22.4 (11.4, 35.6)	18.3 (5.7, 32.2)	6.7 (2.5, 12.3)	7 (2.5, 12.9)
Metabolic risks	69.5 (61, 77.1)	74.9 (66.7, 81.7)	57.1 (45.8, 67.1)	67.7 (55.9, 76.9)	51 (42.3, 59.8)	51.3 (41.9, 60.2)
High LDL cholesterol	32.1 (22.1, 42.2)	31.5 (21.4, 41.9)	8 (2.7, 13.1)	12.7 (4.3, 20.9)	/	/
High systolic blood pressure	40 (30.2, 50)	49.7 (39.9, 59.2)	46.5 (33.4, 58.8)	56 (41.8, 68.1)	11.2 (2, 20.8)	13 (2.5, 23.7)
High fasting plasma glucose	10.3 (8.7, 12)	12.3 (10.5, 14.1)	7 (5.1, 9)	10.2 (7.9, 12.8)	23.5 (17.9, 30.3)	25.8 (19.8, 32.9)
High body mass index	4.5 (1.5, 7.3)	9.4 (3.6, 15.2)	0.4 (−0.1, 1.5)	3.6 (0.3, 7.6)	5.2 (1.7, 13.8)	13.9 (4, 34.3)
Kidney dysfunction	13.5 (9.8, 17)	11.8 (8.3, 15.3)	8.1 (6, 10.4)	7.5 (5.3, 9.8)	28.1 (19.2, 36.9)	24.6 (16.6, 33.5)

LDL, low-density lipoprotein.

## Discussion

This study, utilizing data from the GBD 2021, investigated the epidemiological burden of IHD, stroke, and lower extremity PAD in China from 1990 to 2021. The overall trends of DALYs and incidence rates have shown an upward trajectory over the past 3 decades. As for ASR of incidence, IHD has exhibited a fluctuating upward trend, while stroke has demonstrated a decline. The age-standardized DALYs for IHD have shown a decreasing trend among females and an increasing trend among males. Metabolic risks had the largest population-attributable fraction.

In recent decades, the incidence of IHD has shown varying trends across different regions globally. For instance, a report (1) found that while IHD incidence rates have been decreasing in high-income countries, these trends starkly contrast with those observed in lower-income regions. Among Native Americans, the prevalence of coronary artery disease decreased from 38.6% in 2015 to 36.7% in 2019 (15). In Europe, there are huge differences in ASCVD incidence and mortality between different countries, and ASCVD-related premature deaths under the age of 70 are particularly worrying (16). In low- and middle-income countries, such as the Western Pacific region, the deaths and DALYs of IHD have generally increased, driven by risk factors such as obesity, diabetes, and smoking (17). In some populous countries, such as India, the burden of ASCVD remains persistently high. India accounts for 23.1 and 14% of global DALYs due to IHD and stroke, respectively (18). These contrasting patterns highlight the importance of tailored public health strategies targeting specific regional risks, which could better address the increasing burden of IHD in countries like China.

Stroke and PAD, exhibit distinct patterns of variation. Between 1990 and 2019, the number of global deaths from ischemic stroke increased from 2.04 million annually to 3.29 million (19). In 2019, the global economic loss due to stroke amounted to approximately USD 2,059.67 billion, accounting for 1.66% of the global GDP (20). In China, between 1992 and 2015, the largest increase in the first incidence of stroke was observed among adults aged 55 to 64 years, which is a concerning phenomenon (21). High systolic blood pressure, exposure to environmental particulate pollution, smoking, and a high-sodium diet were the primary attributable risks for the stroke burden in China in 2019 (22). PAD contributes to a relatively smaller burden of DALYs and mortality. Additionally, public awareness of PAD is significantly lower (23). Between 1990 and 2019, the age-standardized global burden related to PAD declined slowly (24). In the United States, lower extremity PAD affects approximately 10% of the population to varying degrees (25). Relatively few studies have examined the burden of lower extremity PAD in China, and our research aims to fill this critical gap.

The gender difference in the burden of ASCVDs was observed. Several studies have identified lifestyle factors, such as smoking and physical inactivity, as predominant contributors to this disparity. For instance, smoking has a negative impact on platelet function, fibrinolysis, inflammation, and vasomotor function, all of which are associated with the development of ASCVD. Differences in tobacco use and tobacco tolerance between genders may contribute to differences in disease burden (26). Additionally, gender differences in metabolic risks such as hypertension and dyslipidemia have been documented (27). Moreover, healthcare access, health-seeking behavior, and biological susceptibility contribute to these observed disparities. The growing evidence underscores the necessity for gender-specific prevention strategies to address the unique disease burden experienced by patients with ASCVD in China.

Assessing the impact of rIsk factors is a crucial prerequisite for reducing the burden of ASCVD (28). Numerous studies have demonstrated the associations between risk factors and ASCVD. For instance, sleep disorder elevates the risk of ASCVD by approximately 8.5 times (29). As for metabolic risks, such as blood lipids, gut microbiota, and blood glucose have causal relationships with the onset of ASCVD (30, 31). Notably, the disease burden attributed to risks exhibits significant regional and gender variations. For instance, household air pollution caused by solid fuels in low-income countries is an attributable risk for DALYs due to ASCVD, and compared to females, males are more likely to be influenced by smoking (32). We identified metabolic risks as the primary source of the disease burden among ASCVD patients, providing a basis for reducing the burden by controlling metabolic risks. Metabolic factors can exacerbate the disease burden of ASCVD by influencing the stability of atherosclerotic plaques. For instance, studies have revealed that poor lipid control can lead to the formation of vulnerable plaques, which is closely associated with an elevated risk of major adverse cardiovascular events, such as mortality, in patients with atherosclerosis (33, 34).

The findings will provide high-quality evidentiary support for the formulation of public health policies related to ASCVD in China. When developing strategies for the prevention and control of relevant diseases, it is essential to take into account the impact of gender and metabolic factors on the disease burden, as well as to incorporate the dynamic trends in the epidemiological burden of the diseases. The strengths of this study include the following. Firstly, the GBD database provides a comprehensive, standardized dataset that aggregates diverse health metrics across countries and regions. This global framework enables cross-regional comparisons, thereby deepening insights into regional disparities and commonalities in disease burden. Secondly, the GBD employs rigorous methodologies for data collection and estimation, incorporating demographic, socioeconomic, and epidemiological data from multiple sources to enhance the reliability of its findings. Conversely, certain limitations must be acknowledged. Firstly, while the GBD offers methods for imputing missing data, the reliance on algorithms and statistical models may not account for local variations or emerging trends effectively. Secondly, the complexity of ASCVD, characterized by multifactorial causation and interactions among various determinants, warrants caution in attributing burden solely based on statistical outputs, as it may overlook critical contextual factors influencing health outcomes.

## Data Availability

Publicly available datasets were analyzed in this study. This data can be found at: https://www.healthdata.org/research-analysis/gbd-data.
