# Supplementary material for: The epidemiology and burden of atherosclerotic cardiovascular disease in China from 1990 to 2021: findings from the global burden of disease 2021
Source: Front Public Health. 2025 Jun 26;13:1529506. doi: 10.3389/fpubh.2025.1529506 (PMC12240750; doi:10.3389/fpubh.2025.1529506)
Supplement: Supplementary file 1 [file Table_1.DOCX]

Supplementary table 1. APCs of joinpoint regression model

| Sex | Model | Segment | Start | End | APC | APC 95% LCL | APC 95% UCL | P-Value |
| --- | --- | --- | --- | --- | --- | --- | --- | --- |
| **Trends in ASR of DALYs for Ischemic Heart Disease** | | | | | |  |  |  |
| General Population | 4 | 0 | 1990 | 1998 | -0.4255 | -0.6518 | -0.1988 | 0.000962 |
| General Population | 4 | 1 | 1998 | 2004 | 3.9906 | 3.6065 | 4.3761 | <0.0001 |
| General Population | 4 | 2 | 2004 | 2007 | -1.8019 | -3.2864 | -0.2947 | 0.021939 |
| General Population | 4 | 3 | 2007 | 2011 | 1.4806 | 0.6045 | 2.3644 | 0.002232 |
| General Population | 4 | 4 | 2011 | 2021 | -1.682 | -1.8872 | -1.4763 | <0.0001 |
| Females | 4 | 0 | 1990 | 1998 | -1.1132 | -1.3861 | -0.8395 | <0.0001 |
| Females | 4 | 1 | 1998 | 2004 | 3.8444 | 3.3783 | 4.3127 | <0.0001 |
| Females | 4 | 2 | 2004 | 2007 | -2.845 | -4.5357 | -1.1243 | 0.002831 |
| Females | 4 | 3 | 2007 | 2011 | 0.5198 | -0.5164 | 1.5668 | 0.307064 |
| Females | 4 | 4 | 2011 | 2021 | -2.1701 | -2.4018 | -1.9377 | <0.0001 |
| Males | 4 | 0 | 1990 | 1999 | 0.4553 | 0.2252 | 0.6859 | 0.000586 |
| Males | 4 | 1 | 1999 | 2004 | 4.8874 | 4.2724 | 5.506 | <0.0001 |
| Males | 4 | 2 | 2004 | 2007 | -1.1624 | -2.8165 | 0.5197 | 0.162725 |
| Males | 4 | 3 | 2007 | 2011 | 2.0844 | 1.0388 | 3.1408 | 0.000527 |
| Males | 4 | 4 | 2011 | 2021 | -1.3935 | -1.6479 | -1.1385 | <0.0001 |
| **Trends in ASR of Incidence for Ischemic Heart Disease** | | | | | | |  |  |
| General Population | 3 | 0 | 1990 | 1995 | -0.2198 | -0.4738 | 0.0348 | 0.086897 |
| General Population | 3 | 1 | 1995 | 2000 | 2.4898 | 2.1176 | 2.8633 | <0.0001 |
| General Population | 3 | 2 | 2000 | 2008 | 1.0197 | 0.8679 | 1.1716 | <0.0001 |
| General Population | 3 | 3 | 2008 | 2021 | -0.3208 | -0.3766 | -0.2649 | <0.0001 |
| Females | 4 | 0 | 1990 | 1995 | -0.3419 | -0.5222 | -0.1613 | 0.000889 |
| Females | 4 | 1 | 1995 | 2000 | 2.6647 | 2.4002 | 2.93 | <0.0001 |
| Females | 4 | 2 | 2000 | 2010 | 0.6004 | 0.5264 | 0.6744 | <0.0001 |
| Females | 4 | 3 | 2010 | 2015 | -0.9467 | -1.1699 | -0.7229 | <0.0001 |
| Females | 4 | 4 | 2015 | 2021 | 0.3852 | 0.2559 | 0.5146 | 0.000007 |
| Males | 3 | 0 | 1990 | 1995 | -0.1662 | -0.484 | 0.1525 | 0.290091 |
| Males | 3 | 1 | 1995 | 2000 | 2.4013 | 1.9441 | 2.8606 | <0.0001 |
| Males | 3 | 2 | 2000 | 2009 | 1.2184 | 1.0687 | 1.3684 | <0.0001 |
| Males | 3 | 3 | 2009 | 2021 | -0.4868 | -0.5626 | -0.4108 | <0.0001 |
| **Trends in ASR of DALYs for Stroke** | | | |  |  |  |  |  |
| General Population | 5 | 0 | 1990 | 1998 | -1.4788 | -1.6035 | -1.3539 | <0.0001 |
| General Population | 5 | 1 | 1998 | 2004 | 0.2721 | 0.0668 | 0.4778 | 0.012778 |
| General Population | 5 | 2 | 2004 | 2007 | -5.443 | -6.1963 | -4.6835 | <0.0001 |
| General Population | 5 | 3 | 2007 | 2010 | -1.6909 | -2.5582 | -0.8158 | 0.000943 |
| General Population | 5 | 4 | 2010 | 2015 | -3.6435 | -3.9736 | -3.3123 | <0.0001 |
| General Population | 5 | 5 | 2015 | 2021 | -1.6123 | -1.88 | -1.3439 | <0.0001 |
| Females | 5 | 0 | 1990 | 1998 | -1.9831 | -2.1044 | -1.8617 | <0.0001 |
| Females | 5 | 1 | 1998 | 2004 | -0.3861 | -0.5844 | -0.1874 | 0.000875 |
| Females | 5 | 2 | 2004 | 2007 | -6.3445 | -7.0522 | -5.6314 | <0.0001 |
| Females | 5 | 3 | 2007 | 2010 | -2.7269 | -3.5491 | -1.8978 | 0.000005 |
| Females | 5 | 4 | 2010 | 2014 | -4.7545 | -5.2269 | -4.2797 | <0.0001 |
| Females | 5 | 5 | 2014 | 2021 | -1.6089 | -1.7904 | -1.4269 | <0.0001 |
| Males | 1 | 0 | 1990 | 2004 | -0.1881 | -0.4595 | 0.084 | 0.167277 |
| Males | 1 | 1 | 2004 | 2021 | -2.5663 | -2.802 | -2.33 | <0.0001 |
| **Trends in ASR of Incidence for Stroke** | | | | |  |  |  |  |
| General Population | 4 | 0 | 1990 | 1994 | 1.0596 | 0.568 | 1.5536 | 0.000253 |
| General Population | 4 | 1 | 1994 | 2006 | -0.3799 | -0.4815 | -0.2783 | <0.0001 |
| General Population | 4 | 2 | 2006 | 2014 | -1.8218 | -2.0306 | -1.6127 | <0.0001 |
| General Population | 4 | 3 | 2014 | 2019 | 1.3958 | 0.8754 | 1.9188 | 0.000023 |
| General Population | 4 | 4 | 2019 | 2021 | -1.5816 | -3.2991 | 0.1663 | 0.073213 |
| Females | 4 | 0 | 1990 | 1994 | 0.4701 | 0.093 | 0.8485 | 0.017333 |
| Females | 4 | 1 | 1994 | 2005 | -0.5689 | -0.6589 | -0.4787 | <0.0001 |
| Females | 4 | 2 | 2005 | 2014 | -2.5762 | -2.7067 | -2.4455 | <0.0001 |
| Females | 4 | 3 | 2014 | 2019 | 2.2036 | 1.7979 | 2.6109 | <0.0001 |
| Females | 4 | 4 | 2019 | 2021 | -1.0855 | -2.4109 | 0.2579 | 0.106387 |
| Males | 1 | 0 | 1990 | 1994 | 1.8046 | 0.3214 | 3.3097 | 0.018772 |
| Males | 1 | 1 | 1994 | 2021 | -0.4484 | -0.5336 | -0.3631 | <0.0001 |
| **Trends in ASR of DALYs for Lower Extremity Peripheral Arterial Diseases** | | | | | | |  |  |
| General Population | 1 | 0 | 1990 | 2001 | 0.1499 | 0.015 | 0.2851 | 0.030721 |
| General Population | 1 | 1 | 2001 | 2021 | -0.5777 | -0.6302 | -0.5252 | <0.0001 |
| Females | 3 | 0 | 1990 | 1995 | 0.8638 | 0.6888 | 1.0392 | <0.0001 |
| Females | 3 | 1 | 1995 | 2002 | -0.3197 | -0.451 | -0.1883 | 0.000053 |
| Females | 3 | 2 | 2002 | 2017 | -0.9192 | -0.9556 | -0.8828 | <0.0001 |
| Females | 3 | 3 | 2017 | 2021 | 0.1868 | -0.0588 | 0.433 | 0.128837 |
| Males | 1 | 0 | 1990 | 2011 | 0.5621 | 0.5021 | 0.6221 | <0.0001 |
| Males | 1 | 1 | 2011 | 2021 | -0.7 | -0.862 | -0.5377 | <0.0001 |
| **Trends in ASR of Incidence for Lower Extremity Peripheral Arterial Diseases** | | | | | | | |  |
| General Population | 3 | 0 | 1990 | 1994 | 0.9803 | 0.8645 | 1.0962 | <0.0001 |
| General Population | 3 | 1 | 1994 | 2007 | 0.1358 | 0.1143 | 0.1574 | <0.0001 |
| General Population | 3 | 2 | 2007 | 2019 | -0.3126 | -0.337 | -0.2881 | <0.0001 |
| General Population | 3 | 3 | 2019 | 2021 | 0.3725 | 0.0116 | 0.7347 | 0.04364 |
| Females | 3 | 0 | 1990 | 1994 | 1.3906 | 1.2694 | 1.512 | <0.0001 |
| Females | 3 | 1 | 1994 | 2006 | -0.0503 | -0.0761 | -0.0245 | 0.000577 |
| Females | 3 | 2 | 2006 | 2018 | -0.3729 | -0.3984 | -0.3473 | <0.0001 |
| Females | 3 | 3 | 2018 | 2021 | 0.318 | 0.1291 | 0.5072 | 0.002117 |
| Males | 3 | 0 | 1990 | 1997 | 0.4118 | 0.3398 | 0.4839 | <0.0001 |
| Males | 3 | 1 | 1997 | 2005 | 0.8239 | 0.7511 | 0.8969 | <0.0001 |
| Males | 3 | 2 | 2005 | 2011 | 0.0842 | -0.0361 | 0.2046 | 0.160344 |
| Males | 3 | 3 | 2011 | 2021 | -0.406 | -0.4479 | -0.364 | <0.0001 |

APC Annual Percentage Change, ASR Age-Standardized Rate, DALYs Disability-Adjusted Life Years.
